# Undifferentiated and Differentiated PC12 Cells Protected by Huprines Against Injury Induced by Hydrogen Peroxide

**DOI:** 10.1371/journal.pone.0074344

**Published:** 2013-09-23

**Authors:** Marta Pera, Pelayo Camps, Diego Muñoz-Torrero, Belen Perez, Albert Badia, M Victoria Clos Guillen

**Affiliations:** 1 Departament de Farmacologia, de Terapèutica i de Toxicologia, Institut de Neurociències, Universitat Autònoma de Barcelona, Bellaterra, Barcelona, Spain; 2 Laboratori de Química Farmacèutica (Unitat Associada al CSIC), Facultat de Farmàcia, Institut de Biomedicina (IBUB), Universitat de Barcelona, Barcelona, Spain; University of Iowa, United States of America

## Abstract

Oxidative stress is implicated in the pathogenesis of neurodegenerative disorders and hydrogen peroxide (H_2_O_2_) plays a central role in the stress. Huprines, a group of potent acetylcholinesterase inhibitors (AChEIs), have shown a broad cholinergic pharmacological profile. Recently, it has been observed that huprine X (HX) improves cognition in non transgenic middle aged mice and shows a neuroprotective activity (increased synaptophysin expression) in 3xTg-AD mice. Consequently, in the present experiments the potential neuroprotective effect of huprines (HX, HY, HZ) has been analyzed in two different *in vitro* conditions: undifferentiated and NGF-differentiated PC12 cells. Cells were subjected to oxidative insult (H_2_O_2_, 200 µM) and the protective effects of HX, HY and HZ (0.01 µM–1 µM) were analyzed after a pre-incubation period of 24 and 48 hours. All huprines showed protective effects in both undifferentiated and NGF-differentiated cells, however only in differentiated cells the effect was dependent on cholinergic receptors as atropine (muscarinic antagonist, 0.1 µM) and mecamylamine (nicotinic antagonist, 100 µM) reverted the neuroprotection action of huprines. The decrease in SOD activity observed after oxidative insult was overcome in the presence of huprines and this effect was not mediated by muscarinic or nicotinic receptors. In conclusion, huprines displayed neuroprotective properties as previously observed in *in vivo* studies. In addition, these effects were mediated by cholinergic receptors only in differentiated cells. However, a non-cholinergic mechanism, probably through an increase in SOD activity, seems to be also involved in the neuroprotective effects of huprines.

## Introduction

Alzheimer's disease (AD) is the most common neurodegenerative disorder and the most prevalent cause of dementia with ageing. The presence of β-amyloid (Aβ)-containing senile plaques and neurofibrillary tangles in the AD brain are the main hallmarks of the disease and are widely believed to be responsible for neuronal degeneration and cell death in this disorder [1]. Although the AD aetiology and pathogenesis are still unknown, several reports point out that excitotoxicity, reduced energy metabolism, mitochondrial dysfunctions, and oxidative stress are very important mechanisms involved in cell death in AD [2]. This hypothesis is supported by the finding that Aβ peptides are associated with free-radical oxidative stress and are the main cause of cellular dysfunctions [3],[4],[5],[6],[7]. As a consequence of that, necrotic and apoptotic processes occur and are the main pathways of cell death in AD [8],[9],[10],[11].

The key symptoms of AD are primarily caused by cholinergic dysfunction, and a significant correlation has been found between a cortical decrease in cholinergic activity and cognitive deterioration (the cholinergic hypothesis). The present approved therapeutic approach is mainly based on increasing cholinergic transmission using cholinesterase inhibitors (ChEI) [12],[13],[14],[15],[16],[17]. However, the therapeutic benefit of such agents is not entirely explained by increasing activity of the cholinergic system, and a large body of evidence shows that ChEI have multiple effects on the central nervous system, some of which could be regarded as broadly neuroprotective [13],[18],[19]. Thus, donepezil, the most widely prescribed AD therapy, markedly decreases lactate dehydrogenase (LDH) release in cortical cells previously exposed to oxygen-glucose deprivation (OGD), and in addition it has a protective effect in gerbils [19]. Similar results have been observed in cell cultures in which neurotoxicity was induced using Aβ protein [20]. It has been suggested that the efficacy of galantamine, a modest AChEI, with allosteric modulator activity on the nicotinic receptor, can be ascribed to its neuroprotective activity mediated by α7 nicotinic receptors [18]. Rivastigmine, another AChEI recently approved, also has a neuroprotective effect although it is completely independent of nicotinic receptors [21]. Consequently, AChEI might be able to act as disease-modifying anti-Alzheimer drugs rather than as mere palliative drugs [22].

It has been demonstrated that huprines, a group of anticholinesterasic drugs obtained by the molecular hybridization of tacrine and (±)-huperzine A, show high selectivity and a potent inhibitory action on AChE in both *in vitro* and *ex vivo* studies [23],[24], an agonistic action on muscarinic and nicotinic receptors [25],[26], and can affect the binding of ligands to the peripheral site of AChE, thereby inhibiting the amyloidogenic process induced by the enzyme [27],[28]. Despite controversial data about the ability of tacrine to exhibit a neuroprotective effect [21],[29], it has been widely demonstrated that huperzine A, the other component of the huprine molecule, shows a neuroprotective activity against different stimuli in several experimental conditions [29],[30]. Interestingly, a recent *in vivo* study has revealed the neuroprotective effect of huprine X, since in 3xTg mice treated with the drug, it significantly increased the synaptophysin content to levels close to those of non-transgenic mice [31] and improved cognition by regulating some neurochemical processes, such as alpha secretases and glycogen synthase kinase 3-beta, in the same transgenic mice [32].

Clonal cell-lines, such as rat pheochromocytoma PC12 cells, provide a useful model system for the investigation of neuronal injury. In contrast with central nervous system primary cultures, which contain many types of nerve cells as well as glial populations, clonal nerve cell-lines yield homogeneous populations, allow an easy manipulation and control over the extracellular milieu, and provide definite advantages over animal experiments. Due to the multitarget pharmacological profile of huprines and because of the importance of oxidative stress in most neurodegenerative diseases, especially in AD, in the present study the potential neuroprotective effects of huprine X (12-amino-3-chloro-9-ethyl-6,7,10,11-tetrahydro-7,11-methanocycloocta[*b*]quinoline hydrochloride), huprine Y (12-amino-3-chloro-6,7,10,11-tetrahydro-9-methyl-7,11-methanocycloocta[*b*]quinoline hydrochloride) and huprine Z (12-amino-3-fluoro-6,7,10,11-tetrahydro-9-methyl-7,11-methanocycloocta[*b*]quinoline hydrochloride) were assessed. For this purpose, undifferentiated and differentiated PC12 cells were subjected to a necrotic insult (hydrogen peroxide), this being the most common process in all degenerative disorders [11],[33].

## Results

### Effect of huprines on cell death induced by H_2_O_2_ in PC12 cells

#### 1. Undifferentiated PC12 cells

**Figure 1:**
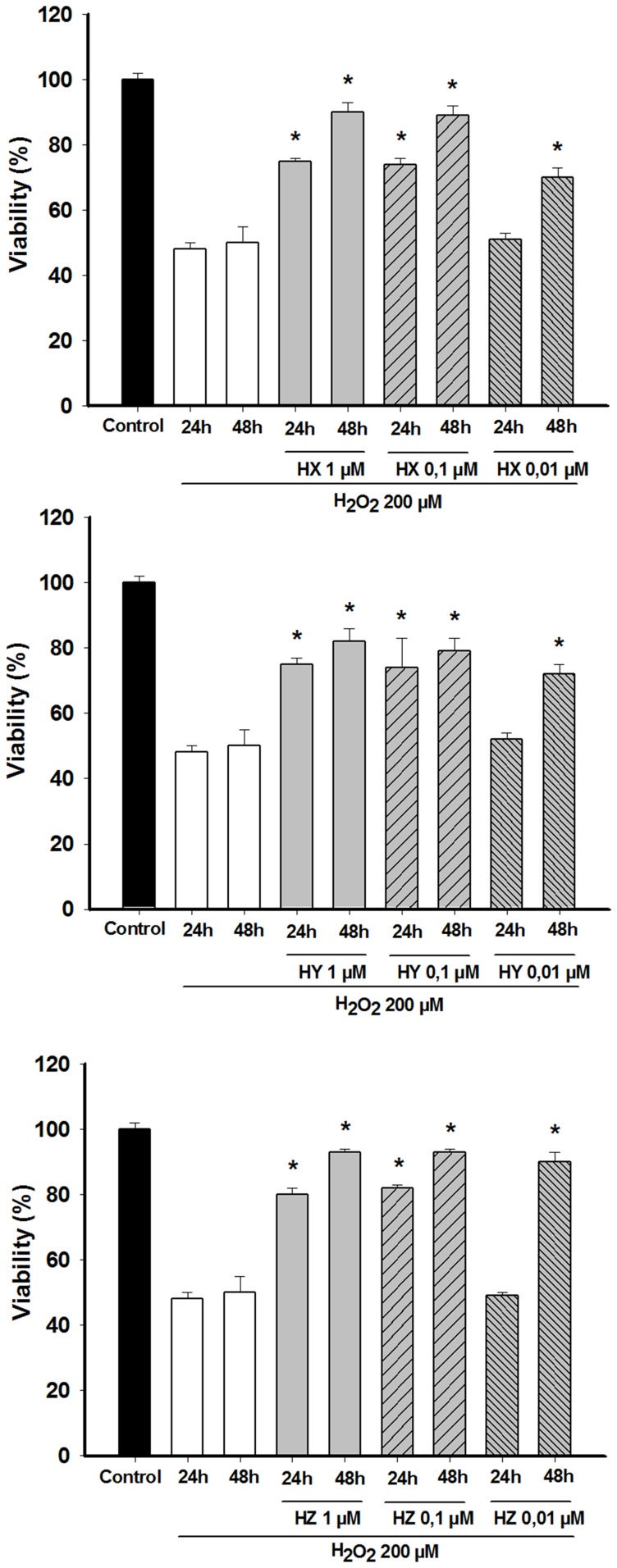
Attenuation of H_2_O_2_-induced cell damage by different concentrations (0.01 µM, 0.1 µM, 1 µM) of (A) HX, (B) HY and (C) HZ in undifferentiated PC12 cells. Cells were incubated with 200 µM H_2_O_2_ for 2 h. Huprines were added to the culture 24–48 h prior to H_2_O_2_ addition. Cell viability was assessed by measuring the MTT reduction. At least three independent experiments were carried out in triplicate. The data are means ± s.e.m. expressed as percentage of control value. *P<0.05 compared with H_2_O_2_ group (Dunnett's test).

Firstly, the cells were incubated in the presence of HX, HY, and HZ (1 µM, 0.1 µM, 0.01 µM) during 1 h and 2 h prior to H_2_O_2_ addition. In these experimental conditions no protective effects were found (data not shown). In the light of these data, we proceeded to increase the incubation time of drugs to 24 h and 48 h prior to H_2_O_2_ addition. The effect of H_2_O_2_ treatment and of the pre-treatment with huprines prior to H_2_O_2_ addition in cell survival is shown in Fig. 1. In the 24 h pre-treatment of huprines prior to H_2_O_2_ addition, drugs induced a percentage of protection between 47% and 65% at 1 µM and 0.1 µM but no significant changes were observed at 0.01 µM (Table 1). When the pre-treatment period was increased to 48 h, a significant increase in the protection percentage was observed at all concentrations used, even at 0.01 µM (between 12% and 70%).

**Table 1:** Effect of huprines pretreatment on undifferentiated PC12 cell survival after exposure of cells to H_2_O_2_ (200 µM).

TREATMENT	% PROTECTION 24 H	% PROTECTION 48 H
Huprine X (1 µM)	47±0.6*	70±4.8*
Huprine X (0.1 µM)	49±2.9*	74±4.6*
Huprine X (0.01 µM)	3±0.9	12±5.4*
Huprine X (1 µM) +MEC (100 µM)		68±0.5^a^
Huprine X (1 µM) +ATR (0.1 µM)		66±10.2^a^
Huprine Y (1 µM)	52±2.3*	60±3.6*
Huprine Y (0.1 µM)	47±8.7*	51±3.3*
Huprine Y (0.01 µM)	4±5.5	22±5.5*
Huprine Y (1 µM) +MEC (100 µM)		61±9.3^b^
Huprine Y (1 µM) +ATR (0.1 µM)		58±1.2^b^
Huprine Z (1 µM)	62±2.2*	65±0.5*
Huprine Z (0.1 µM)	65±1.3*	65±0.5*
Huprine Z (0.01 µM)	0.5±2.4	27±3.7*
Huprine Z (1 µM) +MEC (100 µM)		59±4.7^c^
Huprine Z (1 µM) +ATR (0.1 µM)		55±7.6^c^

Effect of antagonists mecamylamine (MEC) and atropine (ATR) on huprines protective effect on PC12 cells survival after exposure of the cells to H_2_O_2_. See Methods for details of cell treatment and conditions. Values are expressed as percentage of protection (mean ± s.e.m.) obtained from at least three independent experiments run in triplicate. *P<0.05 as compared with H_2_O_2_–treated group (Dunnett's test).^a,b,c^ No significant changes compared with huprine X, Y and Z (1 µM), respectively.

In no case modifications in cell survival were observed when huprines were incubated alone (without H_2_O_2_) at concentrations of 0.01 µM, 0.1 µM, and 1 µM, nevertheless, at concentrations as high as 10 µM and 100 µM, huprines reduced cellular viability (data not shown).

#### 2. NGF differentiated PC12 cells

**Figure 2:**
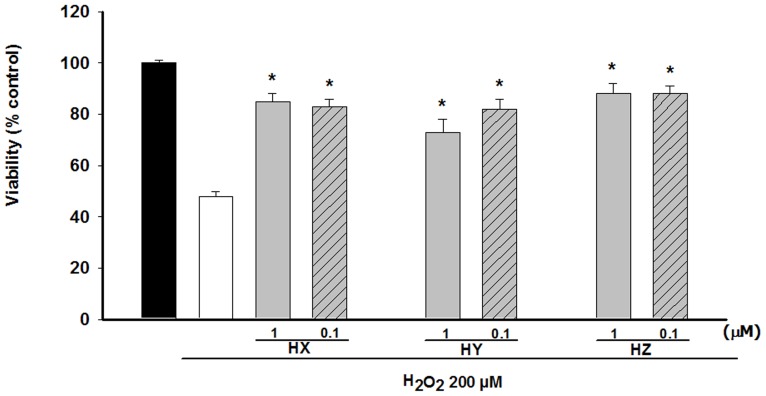
Atenuation of H_2_O_2_-induced cell damage by 0.1 µM and 1 µM concentrations of huprines: HX, HY and HZ in NGF differenciated PC12 cells. Cells were incubated with 200 µM H_2_O_2_ for 2 h. Compounds were added to the culture 48 h prior to H_2_O_2_ addition. Cell viability was assessed by measuring the MTT reduction. At least three independent experiments were carried out in triplicate. The data are means ± s.e.m. expressed as percentage of control value. *P<0.05 compared with H_2_O_2_ group (Dunnett's test).

Similar to undifferentiated PC12 cells, nerve growth factor (NGF)-treated PC12 cells suffered around a 50% reduction of cellular viability when they were exposed to the H_2_O_2_ insult (200 µM) for 2 h. Taking the results obtained with undifferentiated PC12 cells as starting point, in this set of experiments drugs were pre-incubated for 48 h and HX, HY, and HZ were added at concentrations of 0.1 µM and 1 µM (Fig. 2). In this experimental approach a percentage of protection ranging from 44% to 56% was obtained with all of the huprines at both concentrations used (Table 2).

**Table 2:** Effect of huprines pretreatment on NGF differentiated PC12 cells survival after exposure of cells to H_2_O_2_ (200 µM).

TREATMENT	% PROTECTION 48 H
Huprine X (1 µM)	53±5.0*
Huprine X (0.1 µM)	44±6.0*
Huprine X (1 µM) +MEC (100 µM)	7±3.2^b^
Huprine X (1 µM) +ATR (0.1 µM)	32±2.7^b^
Huprine Y (1 µM)	56±5.0*
Huprine Y (0.1 µM)	49±6.1*
Huprine Y (1 µM) +MEC (100 µM)	2.3±0.9^b^
Huprine Y (1 µM) +ATR (0.1 µM)	16±1.6^b^
Huprine Z (1 µM)	56±7.0*
Huprine Z (0.1 µM)	47±7.5*
Huprine Z (1 µM) +MEC (100 µM)	17±3.4^b^
Huprine Z (1 µM) +ATR (0.1 µM)	27±7.1^b^

Effect of antagonists mecamylamine (MEC) and atropine (ATR) on huprines protective effect on PC12 cells survival after exposure of the cells to H_2_O_2_. See Methods for details of cell treatment and conditions. Values are expressed as percentage of protection (mean ± s.e.m.) obtained from at least three independent experiments run in triplicate. *P<0.05 as compared with H_2_O_2_–treated group; ^b^P<0.05 as compared with 1 µM concentration of corresponding drug (Dunnett's test).

### Role of muscarinic and nicotinic receptors in the neuroprotective effects of huprines

#### 1. Undifferentiated PC12 cells

Because previous studies in our laboratory have shown that huprines interact with muscarinic and nicotinic receptors [26],[34],[35], we studied the possible role of cholinergic receptors in the neuroprotective effect exerted by huprines. For this purpose, MEC (100 µM), a nicotinic receptor (nAChR) antagonist, or ATR (0.1 µM), a muscarinic receptor antagonist, were used. Each antagonist was added to the medium concomitantly with huprines. Neither MEC nor ATR antagonised the increase of the survival induced by 48 h of pre-treatment with huprines in undifferentiated PC12 cells (Table 1). These results suggest that the neuroprotective effect of huprines in this cell line is not mediated by an interaction with muscarinic or nicotinic receptors.

Worthy of note, addition of MEC and ATR alone did not modify cell viability.

#### 2. NGF-differentiated PC12 cells

The role of muscarinic and nicotinic receptors in NGF-differentiated PC12 cells was also assessed. In this case, MEC (100 µM) significantly prevented the protective effect of huprines, reducing significantly the neuroprotection percentage induced by drugs (Table 2). ATR (0.1 µM) also significantly reduced the neuroprotection effects of huprines, however this reduction was lower than that observed in the presence of MEC.

### Effect of huprines on SOD activity

#### 1. Undifferentiated PC12 cells

**Figure 3:**
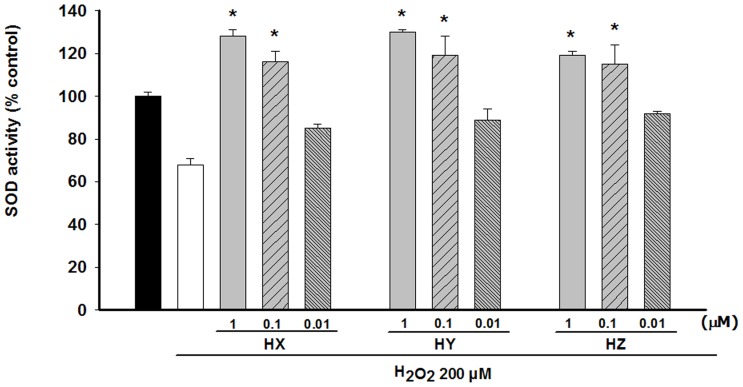
Modulation of the SOD activity in undifferentiated PC12 cells after preincubation with HX, HY and HZ. Cells were incubated with 200 µM H_2_O_2_ for 2 h. Huprines were added to the culture 48 h prior to H_2_O_2_ addition. SOD activity was measured with a SOD activity assay from Fluka. At least three independent experiments were carried out in triplicate. The data are means ± s.e.m. expressed as percentage of control value. *P<0.05 compared with H_2_O_2_ group (Dunnett's test).

PC12 cultures exposure to H_2_O_2_ (200 µM) for 2 h produced a marked decrease of superoxide dismutase (SOD) activity, as compared with control cells. The 48 h pre-treatment of the undifferentiated PC12 cells with HX, HY, and HZ (0.01 µM–1 µM) prior to H_2_O_2_ addition led to a significant increase (24%–86%) of SOD activity, when compared with the H_2_O_2_-treated group (Fig. 3). The levels of SOD activity after the huprines (0.1 µM–1 µM) treatment could also surpass the SOD activity levels of the control cells. This increase in SOD activity might be related to the neuroprotective effect of the huprines against H_2_O_2_.

Incubation of huprines in the absence of H_2_O_2_ did not modify the enzyme activity.

#### 2. NGF-differentiated PC12 cells

**Figure 4:**
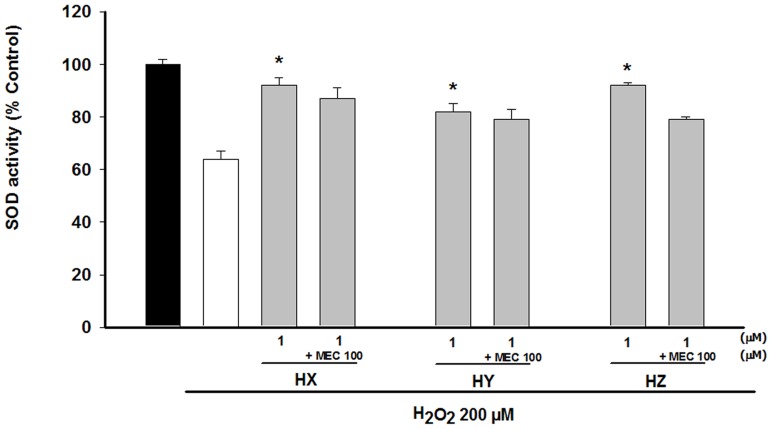
Modulation of the SOD activity in NGF-PC12 differenciated cells by HX, HY and HZ. Huprines were added alone or concomitantly with MEC (100 µM) to the culture 48 h prior to H_2_O_2_ addition. Then, cells were incubated with 200 µM H_2_O_2_ for 2 h. SOD activity was measured using a SOD activity assay from Fluka. At least three independent experiments were carried out in triplicate. The data are means ± s.e.m. expressed as percentage of control value. *p<0.05 compared with H_2_O_2_ group (Dunnett's test).

The 2 h exposure to H_2_O_2_ (200 µM) led to a remarkable reduction in SOD activity, while pre-treatment with huprines prior to H_2_O_2_ addition significantly increased the enzymatic activity. This modification was found in all of the huprines at the concentration of 1 µM (Fig. 4). Given that cell viability is greatly reduced in the presence of MEC in NGF-treated PC12 cells, and the fact that nAChRs have been involved in SOD activation [36], the role of such receptors in the SOD activity was assessed. MEC slightly reversed the enzyme activity, but this reversion was not statistically significant (Fig. 4). ATR did not modify the increase of SOD induced by drugs (data not shown).

## Discussion

Besides the pathological hallmarks of AD, which include the accumulation of protein deposits in the brain as Aβ plaques and neurofibrillary tangles (NFT), AD brains exhibit constant evidence of reactive oxygen species (ROS) - and reactive nitrogen species (RNS)-mediated injury [37]. In some circumstances the production of oxidant species can exceed the endogenous antioxidant ability to destroy them, and an oxidative imbalance occurs. Consequently, the protection of neurons from oxidative damage and death is an important challenge in the development of new treatments against neurodegenerative diseases. PC12 is a cell line characterised by showing sympathetic neuronal cell properties, morphologically, physiologically, and biochemically [38]. In addition, when they are incubated with NGF, PC12 cells increase the expression of cholinergic receptors, choline acetyltransferase expression and acetylcholinesterase activity [39]. Therefore, PC12 cells represent an appropriate experimental approach to study the role of cholinergic receptors in the neuroprotective effect of acetylcholinesterase inhibitors under different conditions.

First of all, we subjected the undifferentiated PC12 cells to an oxidative insult, such as hydrogen peroxide, not only because it is a hydrogen free-radical generator, but also because the toxicity of Aβ, the major pathological hallmark for AD, is partially mediated by hydrogen peroxide [40]. Using this experimental approach, the present results have revealed that HX, HY, and HZ induced a significant protective effect against hydrogen peroxide. All huprines showed a similar effect, suggesting that the slight structural differences among the three compounds are not relevant for their protective activity.

Neuroprotection is a very common feature among different AChEIs in response to different harmful insults. Thus, the acetylcholinesterase inhibitor galantamine showed a protective effect after Aβ or thapsigargin stimulus in the human neuroblastoma cell line SH-SY5Y, as well as in bovine chromaffin cells [41]. Additionally, donepezil, rivastigmine and galantamine showed a neuroprotective effect against okadaic acid in undifferentiated SH-SY5Y cells [21]. Huperzine A, one of the parent compounds from which huprines were designed, showed a protective effect after inducing oxygen-glucose deprivation in pheochromocytoma cells, most likely by alleviating disturbances of oxidative and energy metabolism [42] or in the presence of hydrogen peroxide, which improved expression of apoptosis-related genes [43]. Nevertheless, it is necessary to state that the protective effect of huprines was only apparent when cells were preincubated with the drugs for periods of 24 h or longer (48 h), suggesting that a prolonged treatment before hydrogen peroxide exposure is necessary to elicit their neuroprotective activity. Indeed, when drugs were added concomitantly with hydrogen peroxide or even a few hours before inducing the insult, no protective effects of huprines were observed (data not shown). The relevance of the incubation time prior to the addition of some toxic agents is well established in the literature. Studies performed with donepezil, galantamine, and tacrine in cortical neurons using glutamate as a toxic agent have shown that the protective effects of these drugs were more pronounced at longer incubation periods [44],[45]. It has been suggested that a delay in the neuroprotective effects of some AChEIs could be ascribed to the cascade of events that take place after nicotinic receptor activation [44]. Herein, the neuroprotective effects of huprines were higher when the preincubation time was extended up to 48 h, and in these conditions the effects of the drugs were also observed at a concentration as low as 10 nM. These data could also suggest that the effects of huprines are less apparent when the cell damage has been already established. In fact, HX improved cognitive deficits in 3xTg-AD mice 7 months old [41] but such effect was not observed in 12 month old 3xTg-AD mice (non published data).

In addition to their high affinity inhibition of AChE (picomolar range) these compounds are able to stimulate muscarinic and nicotinic receptors [25],[34],[46]. These data led us to speculate that the agonistic effect of the huprines on cholinergic receptors could trigger the neuroprotective effect together with the potentiation of the cholinergic system associated with the inhibitory effect on AChE. In fact, the neuroprotective effect observed in some AChEIs has been ascribed to their activity on nicotinic receptors [18]. In order to analyse the possible involvement of cholinergic receptors in the protection induced by huprines, MEC and ATR were used to block nicotinic and muscarinic receptors, respectively. Both antagonists failed to reverse the effect of all compounds in undifferentiated cells, suggesting that the protection induced by huprines was not related to their activity on cholinergic receptors. The latency period between the addition of drugs to the incubation medium and detection of the protective effect seems to indicate that the mechanism of these drugs might include a chain of events inducing the activation/inactivation of different factors.

Hydrogen peroxide is a free-radical generator that induces cell and tissue damage. SOD is an antioxidant enzyme that plays a pivotal role in maintaining a very low, steady state of intracellular O_2_^-^ [47]. The activity of this enzyme was significantly increased by huprines in undifferentiated cells, suggesting that changes in SOD expression and/or activity might be involved in the protective effects of huprines.

It is known that exposure of PC12 cells to NGF leads to changes in their properties, increasing the cholinergic receptors expression. In a second set of experiments we subjected PC12 cells to the presence of NGF and once they were differentiated, cells were treated for 48 h with huprines. As it was observed in undifferentiated cells, HX, HY, and HZ elicited a significant protective effect after adding hydrogen peroxide to the culture medium; however the percentage of protection was lower than in the case of undifferentiated cells. Differentiated cells seem to be more sensitive to oxidant stimulus than undifferentiated cells [48], however, in the present study all cells showed a similar sensitivity to hydrogen peroxide. Huprines were not able to restore cell viability with the same efficacy than in undifferentiated cells.

As mentioned above, NGF-differentiated PC12 cells increase the presence of cholinergic factors, especially nicotinic and muscarinic receptors, therefore cells were incubated with huprines and concomitantly with MEC or ATR to block nicotinic and muscarinic receptors, respectively. In contrast to that observed in undifferentiated cells, in NGF-differentiated PC12 cells a significant reduction of the neuroprotective effects of huprines was observed when the antagonists were added, achieving survival values similar to those obtained in non-treated cells, especially in the presence of MEC. ATR only induced a partial, but significant reversion, of the protective effects of huprines. The role of nicotinic receptors in neuroprotection has been widely described. The higher transcription of nAChR, and especially the α5, α7, and β4 subunits of the nAChR in NGF-differentiated cells [49], might explain the differences obtained in undifferentiated and NGF-differentiated PC12 cells. It has also been shown that the neuroprotective effect of donepezil and galantamine against glutamate could be reverted by α7 and β4 nAChR antagonists and by some inhibitors of the IP3-kinases pathway [50]. This complex pathway implies changes in a large number of proteins involved in cellular proliferation and/or apoptosis, modifying the balance between these two processes. Our data suggest that the protection induced by huprines could be mainly related to the nAChRs in NGF-differentiated cells. The higher reduction of the neuroprotective effects of the huprines in the presence of the nicotinic antagonist, as compared with the muscarinic antagonist, is in consonance with the results obtained in other studies, which highlight the role of nAChR in AD development [44],[50]. However, muscarinic receptors have also been related to neuroprotection [51],[52]. Thus, activation of muscarinic receptors could increase the antiapoptotic protein Bcl2 via MAPK, thus preserving the viability of mouse cerebellar granule cells [53]. Herein, the effects of huprines on cell viability were also partially mediated by muscarinic receptors in NGF-differentiated cells.

Finally, the activity of SOD in NGF-differentiated cells was also analysed. As in the case of undifferentiated PC12 cells, a decrease of SOD activity was induced by hydrogen peroxide. Again, the presence of huprines promoted the recovery of enzyme activity. *In vivo* administration of galantamine, an AChEI with nicotinic activity, provides neuroprotection to gerbils in an ischaemia model increasing, among others, SOD activity, and this effect was inhibited by MEC [36]. Rivastigmine and also huperzine A and huperzine B increase SOD activity [54],[55],[56], however, none of these drugs has affinity to nAChRs. It has been demonstrated that nAChRs modulate SOD activity by the activation of PI3K/Akt, nevertheless, the same authors have highlighted the complex regulation of this enzyme, suggesting that multiple positive and negative regulatory elements can be involved [57]. In order to rule out the role of nAChRs in the increase of SOD activity induced by huprines, MEC was also tested in NGF-differentiated cells. Antagonist MEC was unable to modify the effect of huprines on SOD, suggesting that the increase of the enzyme activity induced by huprines is not directly mediated by cholinergic receptors.

In summary, HX, HY, and HZ have shown neuroprotective effects, thereby supporting previous data observed in *in vivo* studies [31]. In addition, the subtle structural differences among huprines seem to be not relevant for the neuroprotective effect, as all of them provided similar neuroprotective activity. In addition, the protection against hydrogen peroxide was independent of cholinergic receptors in undifferentiated cells, whereas in NGF-differentiated cells nAChRs seem to be involved in this effect, as MEC was able to reverse the effects of huprines. Finally, huprines increased SOD activity, which suggests the ability of the drugs to decrease ROS accumulation in response to oxidative stress. The fact that huprines induced their protective effect by different mechanisms in differentiated cells, as compared to undifferentiated ones, and that their action on SOD activity was totally independent of nicotinic receptors, indicates that multiple pathways can contribute to the protective activity of drugs, and cellular mechanisms involved in such processes are also dependent on the cellular system and environmental conditions in which they are studied. These *in vitro* results could support *in vivo* data observed in NTg and 3xT-AD mice treated by HX in which an improvement in cognition and a positive regulation of synaptophysin have been described [31],[32]. Consequently, more experiments must be carried out to shed more light on the molecular mechanisms involved in the neuroprotective effect of huprines.

## Material and Methods

### Materials

3-(4,5-Dimethylthiazol-2-yl)-2,5-diphenyltetrazolium bromide (MTT), dimethyl sulfoxide (DMSO), EDTA, foetal bovine serum, glutamine, HEPES, horse serum, hydrogen peroxide, nerve growth factor β (NGF-β), penicillin, poli-L-lysine hydrobromide, potassium chloride, potassium phosphate monobasic, protease inhibitor cocktail, RPMI-1640, sodium chloride, sodium phosphate dibasic dihydrate, sodium pyruvate, and streptomycin were purchased from Sigma-Aldrich (St. Louis, USA). Glucose was purchased from Merck (Darmstadt, Germany) and the enzyme tripsine-0.2 g EDTA from Sigma-Aldrich (Saint Louis, USA). Huprines X, Y, and Z (HX, HY and HZ) were synthesized at the Laboratori de Química Farmacèutica, Facultat de Farmàcia, Universitat de Barcelona. The antagonists atropine (ATR) and mecamylamine (MEC) hydrochloride were obtained from Sigma-Aldrich (St. Louis, USA). Superoxide dismutase activity was determined using the SOD assay kit from Fluka, Sigma-Aldrich, and the Bradford protein assay from Bio-rad (Hercules, California, USA) was used to quantify proteins.

### Cell culture and treatment

The rat pheochromocytoma cell line PC12 is a classic *in vitro* neuroendocrine cell model. Unlike primary neurons, undifferentiated PC12 cells do not require NGF for survival, but they respond to it by producing lengthy neurite extensions and by undergoing other neural-specific changes such as an increase of cholinergic receptors expression [58],[59]. NGF-treated PC12 cells exhibit many of the hallmarks of differentiated neurons.

PC12 cells (ATCC, CRL-1721) were generously provided by Dr. N. Gomez (Biochemistry and Molecular Biology Department, UAB, Barcelona, Spain) and grown on polystyrene tissue-culture dishes in RPMI-1640 containing 10% horse serum and 5% foetal bovine serum, supplemented with 2 mM glutamine, 1 mM pyruvate, 100 unit/mL penicillin and 100 mg/mL streptomycin at 37°C with 95% air−5% CO_2_. All experiments were carried out 24 h after cells were seeded in 24-well or 6-well plates previously treated with poly-L-lysine hydrobromide. The cellular density in 6-well plates was 200,000 cells/mL and in 24-well plates about 30,000 cells/mL. For experiments in undifferentiated cells 24-well plates were used and the drug treatments were directly started 24 h after seeding the cells. For cell differentiation, the 6-well plates were used and cells were treated with NGF-β 100 ng/mL for 7 days prior to huprines addition. To induce necrosis, cells were incubated with the indicated concentration of H_2_O_2_ for 2 h. To study the effects of huprines on PC12 cells, cells were preincubated with different concentrations of drugs for 24 h or 48 h before addition H_2_O_2_. When antagonists mecamylamine (MEC, nicotinic receptor antagonist) and atropine (ATR, muscarinic receptor antagonist) were used they were added concomitantly with agonists.

### Cell viability assay

Cell viability was measured by the method of MTT described by Mosmann [60]. Briefly, cells in 24-well plates were rinsed with phosphate-buffered saline (PBS), MTT (0.5 mg/mL) was added to each well and incubated for 30 min at 37°C. After removal of the medium with MTT, cells and dye crystals were solubilised with 200 µL DMSO, and optical density was measured at 570 nm on a microplate reader. The percentage of protection was calculated according to the following equation:
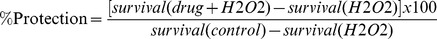



### Enzymatic assay

For the assay of SOD activity, the cultures were washed with ice-cold PBS and then pooled in 0.1 M PBS, 0.05 mM EDTA buffered solution and homogenized. The homogenate was centrifuged for 1 h at 10,000xg. The resulting supernatants were used in this assay. Superoxide dismutase activity was determined using the SOD assay Kit-WST, obtained from Fluka and was measured in accordance with the instructions supplied by the manufacturer. Briefly, samples were incubated at 37°C for 20 min and the SOD activity, as an inhibition activity, was quantified by measuring the decrease in the color development (amount of superoxide anion) at 440 nm. The effect of huprines on SOD activity was evaluated as the percentage of control group values. The protein level in cells was measured by Bradford method, using bovine serum albumin as standard [61].

### Statistical analysis

Data are expressed as means ± SEM and evaluated for statistical significance (P<0.05) with one-way ANOVA followed by Duncan's multiple-range test.

## References

[1] Mucke L (2009) Neuroscience: Alzheimer's disease. Nature 461: 895–897. 19829367 10.1038/461895a

[2] Mehta A, Prabhakar M, Kumar P, Deshmukh R, Sharma PL (2013) Excitotoxicity: bridge to various triggers in neurodegenerative disorders. Eur J Pharmacol 698: 6–18. 23123057 10.1016/j.ejphar.2012.10.032

[3] Markesbery WR, Carney JM (1999) Oxidative alterations in Alzheimer's disease. Brain Pathol 9: 133–146. 9989456 10.1111/j.1750-3639.1999.tb00215.x PMC8098393

[4] Butterfield DA, Hensley K, Harris M, Mattson M, Carney J (1994) beta-Amyloid peptide free radical fragments initiate synaptosomal lipoperoxidation in a sequence-specific fashion: implications to Alzheimer's disease. Biochem Biophys Res Commun 200: 710–715. 8179604 10.1006/bbrc.1994.1508

[5] Butterfield DA (1997) beta-Amyloid-associated free radical oxidative stress and neurotoxicity: implications for Alzheimer's disease. Chem Res Toxicol 10: 495–506. 9168246 10.1021/tx960130e

[6] Bennett S, Grant MM, Aldred S (2009) Oxidative stress in vascular dementia and Alzheimer's disease: a common pathology. J Alzheimers Dis 17: 245–257. 19221412 10.3233/JAD-2009-1041

[7] Jomova K, Vondrakova D, Lawson M, Valko M (2010) Metals, oxidative stress and neurodegenerative disorders. Mol Cell Biochem 345: 91–104. 20730621 10.1007/s11010-010-0563-x

[8] Behl C, Davis JB, Lesley R, Schubert D (1994) Hydrogen peroxide mediates amyloid beta protein toxicity. Cell 77: 817–827. 8004671 10.1016/0092-8674(94)90131-7

[9] Zhu X, Raina AK, Perry G, Smith MA (2006) Apoptosis in Alzheimer disease: a mathematical improbability. Curr Alzheimer Res 3: 393–396. 17017869 10.2174/156720506778249470

[10] Gorman AM (2008) Neuronal cell death in neurodegenerative diseases: recurring themes around protein handling. J Cell Mol Med 12: 2263–2280. 18624755 10.1111/j.1582-4934.2008.00402.x PMC4514105

[11] Yamashima T (2013) Reconsider Alzheimer's disease by the 'calpain-cathepsin hypothesis'-A perspective review. Prog Neurobiol. 10.1016/j.pneurobio.2013.02.004 23499711

[12] Bianchetti A, Ranieri P, Margiotta A, Trabucchi M (2006) Pharmacological treatment of Alzheimer's Disease. Aging Clin Exp Res 18: 158–162. 16702787 10.1007/BF03327433

[13] Francis PT, Nordberg A, Arnold SE (2005) A preclinical view of cholinesterase inhibitors in neuroprotection: do they provide more than symptomatic benefits in Alzheimer's disease? Trends Pharmacol Sci 26: 104–111. 15681028 10.1016/j.tips.2004.12.010

[14] Giacobini E (2000) Cholinesterase inhibitor therapy stabilizes symptoms of Alzheimer disease. Alzheimer Dis Assoc Disord 14 Suppl 1S3–10. 10850724 10.1097/00002093-200000001-00002

[15] Martorana A, Esposito Z, Koch G (2010) Beyond the cholinergic hypothesis: do current drugs work in Alzheimer's disease? CNS Neurosci Ther 16: 235–245. 20560995 10.1111/j.1755-5949.2010.00175.x PMC6493875

[16] Akaike A, Takada-Takatori Y, Kume T, Izumi Y (2010) Mechanisms of neuroprotective effects of nicotine and acetylcholinesterase inhibitors: role of alpha4 and alpha7 receptors in neuroprotection. J Mol Neurosci 40: 211–216. 19714494 10.1007/s12031-009-9236-1

[17] Schneider LS (2013) Alzheimer disease pharmacologic treatment and treatment research. Continuum (Minneap Minn) 19: 339–357. 23558481 10.1212/01.CON.0000429180.60095.d0 PMC10564039

[18] Kita Y, Ago Y, Takano E, Fukada A, Takuma K, et al (2013) Galantamine increases hippocampal insulin-like growth factor 2 expression via alpha7 nicotinic acetylcholine receptors in mice. Psychopharmacology (Berl) 225: 543–551. 22932776 10.1007/s00213-012-2841-7

[19] Min D, Mao X, Wu K, Cao Y, Guo F, et al (2012) Donepezil attenuates hippocampal neuronal damage and cognitive deficits after global cerebral ischemia in gerbils. Neurosci Lett 510: 29–33. 22240104 10.1016/j.neulet.2011.12.064

[20] Kimura M, Akasofu S, Ogura H, Sawada K (2005) Protective effect of donepezil against Abeta(1–40) neurotoxicity in rat septal neurons. Brain Res 1047: 72–84. 15893738 10.1016/j.brainres.2005.04.014

[21] Arias E, Gallego-Sandin S, Villarroya M, Garcia AG, Lopez MG (2005) Unequal neuroprotection afforded by the acetylcholinesterase inhibitors galantamine, donepezil, and rivastigmine in SH-SY5Y neuroblastoma cells: role of nicotinic receptors. J Pharmacol Exp Ther 315: 1346–1353. 16144975 10.1124/jpet.105.090365

[22] Racchi M, Mazzucchelli M, Porrello E, Lanni C, Govoni S (2004) Acetylcholinesterase inhibitors: novel activities of old molecules. Pharmacol Res 50: 441–451. 15304241 10.1016/j.phrs.2003.12.027

[23] Badia A, Baños JE, Camps P, Contreras J, Görbig DM, et al (1998) Synthesis and evaluation of tacrine-huperzine A hybrids as acetylcolinesterase inhibitors of potential interest for the treatment of Alzheimer's disease. Bioorg Med Chem 6: 427–440. 9597187 10.1016/s0968-0896(98)00015-7

[24] Camps P, El Achab R, Morral J, Muñoz-Torrero D, Badia A, et al (2000) New tacrine-huperzine A hybrids (huprines): highly potent tight-binding acetylcholinesterase inhibitors of interest for the treatment of Alzheimer's disease. J Med Chem 43: 4657–4666. 11101357 10.1021/jm000980y

[25] Roman S, Vivas NM, Badia A, Clos MV (2002) Interaction of a new potent anticholinesterasic compound (+/−)huprine X with muscarinic receptors in rat brain. Neurosci Lett 325: 103–106. 12044632 10.1016/s0304-3940(02)00245-8

[26] Roman S, Badia A, Camps P, Clos MV (2004) Potentiation effects of (+/−)huprine X, a new acetylcholinesterase inhibitor, on nicotinic receptors in rat cortical synaptosomes. Neuropharmacology 46: 95–102. 14654101 10.1016/j.neuropharm.2003.08.005

[27] Pera M, Roman S, Ratia M, Camps P, Munoz-Torrero D, et al (2006) Acetylcholinesterase triggers the aggregation of PrP 106–126. Biochem Biophys Res Commun 346: 89–94. 16750169 10.1016/j.bbrc.2006.04.187

[28] Pera M, Martinez-Otero A, Colombo L, Salmona M, Ruiz-Molina D, et al (2009) Acetylcholinesterase as an amyloid enhancing factor in PrP82–146 aggregation process. Mol Cell Neurosci 40: 217–224. 19038345 10.1016/j.mcn.2008.10.008

[29] Xiao XQ, Wang R, Tang XC (2000) Huperzine A and tacrine attenuate beta-amyloid peptide-induced oxidative injury. J Neurosc Res 61: 564–9. 10.1002/1097-4547(20000901)61:5<564::AID-JNR11>3.0.CO;2-X 10956426

[30] Wang R, Yan H, Tang XC (2006) Progress in studies of huperzine A, a natural cholinesterase inhibitor from Chinese herbal medicine. Acta Pharmacol Sin 27: 1–26. 10.1111/j.1745-7254.2006.00255.x 16364207

[31] Hedberg MM, Clos MV, Ratia M, Gonzalez D, Lithner CU, et al (2010) Effect of huprine X on beta-amyloid, synaptophysin and alpha7 neuronal nicotinic acetylcholine receptors in the brain of 3xTg-AD and APPswe transgenic mice. Neurodegener Dis 7: 379–388. 20689242 10.1159/000287954

[32] Ratia M, Gimenez-Llort L, Camps P, Muñoz-Torrero D, Perez B, et al (2013) Huprine X and huperzine A improve cognition and regulate some neurochemical processes related with Alzheimer's disease in triple transgenic mice (3xTg-AD). Neurodegener Dis 11: 129–140. 22626981 10.1159/000336427

[33] Stadelmann C, Bruck W, Bancher C, Jellinger K, Lassmann H (1998) Alzheimer disease: DNA fragmentation indicates increased neuronal vulnerability, but not apoptosis. J Neuropathol Exp Neurol 57: 456–464. 9596416 10.1097/00005072-199805000-00009

[34] Alcala MM, Maderuelo A, Vivas NM, Camps P, Muñoz-Torrero D, et al (2005) Effects of (+/−)-huprine Y and (+/−)-huprine Z, two new anticholinesterasic drugs, on muscarinic receptors. Neurosci Lett 379: 106–109. 15823425 10.1016/j.neulet.2004.12.044

[35] Roman S, Vivas NM, Badia A, Clos MV (2002) Interaction of a new potent anticholinesterasic compound (+/−)huprine X with muscarinic receptors in rat brain. Neurosci Lett 325: 103–106. 12044632 10.1016/s0304-3940(02)00245-8

[36] Lorrio S, Sobrado M, Arias E, Roda JM, Garcia AG, et al (2007) Galantamine postischemia provides neuroprotection and memory recovery against transient global cerebral ischemia in gerbils. J Pharmacol Exp Ther 322: 591–599. 17526807 10.1124/jpet.107.122747

[37] Pratico D, Sung S (2004) Lipid peroxidation and oxidative imbalance: early functional events in Alzheimer's disease. J Alzheimers Dis 6: 171–175. 15096701 10.3233/jad-2004-6209

[38] Tischler AS, Greene LA (1975) Nerve growth factor-induced process formation by cultured rat pheochromocytoma cells. Nature 258: 341–342. 1196362 10.1038/258341a0

[39] Greene LA, Rukenstein A (1981) Regulation of acetylcholinesterase activity by nerve growth factor. Role of transcription and dissociation from effects on proliferation and neurite outgrowth. J Biol Chem 256: 6363–6367. 7240210

[40] Huang X, Atwood CS, Hartshorn MA, Multhaup G, Goldstein LE, et al (1999) The A beta peptide of Alzheimer's disease directly produces hydrogen peroxide through metal ion reduction. Biochemistry 38: 7609–7616. 10386999 10.1021/bi990438f

[41] Arias E, Ales E, Gabilan NH, Cano-Abad MF, Villarroya M, et al (2004) Galantamine prevents apoptosis induced by beta-amyloid and thapsigargin: involvement of nicotinic acetylcholine receptors. Neuropharmacology 46: 103–114. 14654102 10.1016/s0028-3908(03)00317-4

[42] Zhou J, Fu Y, Tang XC (2001) Hiperzine A protects rat pheochromocytoma cells against oxygen-glucose deprivation. NeuroReport 12: 2073–2077. 11447310 10.1097/00001756-200107200-00007

[43] Wang R, Xiao XQ, Tang XC (2001) Huperzine A attenuates hydrogen peroxide induced apoptosis by regulating expression of apoptosis-related genes in rat PC12 cells. Neuroreport 12: 2629–2634. 11522938 10.1097/00001756-200108280-00009

[44] Takada Y, Yonezawa A, Kume T, Katsuki H, Kaneko S, et al (2003) Nicotinic acetylcholine receptor-mediated neuroprotection by donepezil against glutamate neurotoxicity in rat cortical neurons. J Pharmacol Exp Ther 306: 772–777. 12734391 10.1124/jpet.103.050104

[45] Takada-Takatori Y, Kume T, Sugimoto M, Katsuki H, Sugimoto H, et al (2006) Acetylcholinesterase inhibitors used in treatment of Alzheimer's disease prevent glutamate neurotoxicity via nicotinic acetylcholine receptors and phosphatidylinositol 3-kinase cascade. Neuropharmacology 51: 474–486. 16762377 10.1016/j.neuropharm.2006.04.007

[46] Roman S, Badia A, Camps P, Muñoz-Torrero D, Clos MV (2005) Nicotinic-receptor potentiator drugs, huprine X and galantamine, increase ACh release by blocking AChE activity but not acting on nicotinic receptors. Brain Res 1061: 73–79. 16248990 10.1016/j.brainres.2005.07.042

[47] Zelko IN, Mariani TJ, Folz RJ (2002) Superoxide dismutase multigene family: a comparison of the CuZn-SOD (SOD1), Mn-SOD (SOD2), and EC-SOD (SOD3) gene structures, evolution, and expression. Free Radic Biol Med 33: 337–349. 12126755 10.1016/s0891-5849(02)00905-x

[48] Nouspikel T (2007) DNA repair in differentiated cells: some new answers to old questions. Neuros 145: 1213–1221. 10.1016/j.neuroscience.2006.07.006 16920273

[49] Nakamura S, Takahashi T, Yamashita H, Kawakami H (2001) Nicotinic acetylcholine receptors and neurodegenerative disease. Alcohol 24: 79–81. 11522426 10.1016/s0741-8329(01)00150-1

[50] Takada-Takatori Y, Kume T, Sugimoto M, Katsuki H, Niidome T, et al (2006) Neuroprotective effects of galanthamine and tacrine against glutamate neurotoxicity. Eur J Pharmacol 549: 19–26. 16996497 10.1016/j.ejphar.2006.08.017

[51] Almasieh M, Zhou Y, Kelly ME, Casanova C, Di PA (2010) Structural and functional neuroprotection in glaucoma: role of galantamine-mediated activation of muscarinic acetylcholine receptors. Cell Death Dis 1: e27. 21364635 10.1038/cddis.2009.23 PMC3032334

[52] Picconi B, Barone I, Pisani A, Nicolai R, Benatti P, et al (2006) Acetyl-L-carnitine protects striatal neurons against in vitro ischemia: the role of endogenous acetylcholine. Neuropharmacology 50: 917–923. 16500685 10.1016/j.neuropharm.2006.01.002

[53] Giordano G, Li L, White CC, Farin FM, Wilkerson HW, et al (2009) Muscarinic receptors prevent oxidative stress-mediated apoptosis induced by domoic acid in mouse cerebellar granule cells. J Neurochem 109: 525–538. 19200344 10.1111/j.1471-4159.2009.05969.x PMC4045406

[54] Kumar P, Kumar A (2009) Protective effect of rivastigmine against 3-nitropropionic acid-induced Huntington's disease like symptoms: possible behavioural, biochemical and cellular alterations. Eur J Pharmacol 615: 91–101. 19445928 10.1016/j.ejphar.2009.04.058

[55] Shang YZ, Ye JW, Tang XC (1999) Improving effects of huperzine A on abnormal lipid peroxidation and superoxide dismutase in aged rats. Zhongguo Yao Li Xue Bao 20: 824–828. 11245091

[56] Wang ZF, Zhou J, Tang XC (2002) Huperzine B protects rat pheochromocytoma cells against oxygen-glucose deprivation-induced injury. Acta Pharmacol Sin 23: 1193–1198. 12466060

[57] Rojo AI, Salinas M, Martin D, Perona R, Cuadrado A (2004) Regulation of Cu/Zn-superoxide dismutase expression via the phosphatidylinositol 3 kinase/Akt pathway and nuclear factor-kappaB. J Neurosci 24: 7324–7334. 15317858 10.1523/JNEUROSCI.2111-04.2004 PMC6729771

[58] Jumblatt JE, Tischler AS (1982) Regulation of muscarinic ligand binding sites by nerve growth factor in PC12 phaeochromocytoma cells. Nature 297: 152–154. 7078629 10.1038/297152a0

[59] Amy CM, Bennett EL (1983) Increased sodium ion conductance through nicotinic acetylcholine receptor channels in PC12 cells exposed to nerve growth factors. J Neurosci 3: 1547–1553. 6308184 10.1523/JNEUROSCI.03-08-01547.1983 PMC6564533

[60] Mosmann T (1983) Rapid colorimetric assay for cellular growth and survival: application to proliferation and cytotoxicity assays. J Immunol Methods 65: 55–63. 6606682 10.1016/0022-1759(83)90303-4

[61] Bradford M (1976) A rapid and sensitive method for the quantitation of microgram quantities of protein utilizing the principle of protein-dye binding. Anal Biochem 248: 248–254. 10.1016/0003-2697(76)90527-3 942051

